# Attention Deficit/Hyperactivity Disorder: A Focused Overview for Children’s Environmental Health Researchers

**DOI:** 10.1289/ehp.1002326

**Published:** 2010-09-09

**Authors:** Andréa Aguiar, Paul A. Eubig, Susan L. Schantz

**Affiliations:** 1 Department of Comparative Biosciences, College of Veterinary Medicine and; 2 Neuroscience Program, University of Illinois at Urbana-Champaign, Urbana, Illinois, USA

**Keywords:** ADHD, attention, executive function

## Abstract

**Objectives:**

Attention deficit/hyperactivity disorder (ADHD) is the most frequently diagnosed childhood neurobehavioral disorder. Much research has been done to identify genetic, environmental, and social risk factors for ADHD; however, we are still far from fully understanding its etiology. In this review we provide an overview of diagnostic criteria for ADHD and what is known about its biological basis. We also review the neuropsychological functions that are affected in ADHD. The goal is to familiarize the reader with the behavioral deficits that are hallmarks of ADHD and to facilitate comparisons with neurobehavioral deficits associated with environmental chemical exposures.

**Data sources:**

Relevant literature on ADHD is reviewed, focusing in particular on meta-analyses conducted between 2004 and the present that evaluated associations between measures of neuropsychological function and ADHD in children. Meta-analyses were obtained through searches of the PubMed electronic database using the terms “ADHD,” “meta-analysis,” “attention,” “executive,” and “neuropsychological functions.” Although meta-analyses are emphasized, nonquantitative reviews are included for particular neuropsychological functions where no meta-analyses were available.

**Data synthesis:**

The meta-analyses indicate that vigilance (sustained attention), response inhibition, and working memory are impaired in children diagnosed with ADHD. Similar but somewhat less consistent meta-analytic findings have been reported for impairments in alertness, cognitive flexibility, and planning. Additionally, the literature suggests deficits in temporal information processing and altered responses to reinforcement in children diagnosed with ADHD. Findings from brain imagining and neurochemistry studies support the behavioral findings.

**Conclusions:**

Behavioral, neuroanatomical, and neurochemical data indicate substantial differences in attention and executive functions between children diagnosed with ADHD and non-ADHD controls. Comparisons of the neurobehavioral deficits associated with ADHD and those associated with exposures to environmental chemicals may help to identify possible environmental risk factors for ADHD and/or reveal common underlying biological mechanisms.

In recent years, there has been increasing awareness of the role of environmental factors in neurodevelopmental disorders, including attention deficit/hyperactivity disorder (ADHD) (e.g., Banerjee et al. 2007; Nigg 2006b; Swanson et al. 2007). In this review we provide a focused overview of ADHD for researchers who are interested in the association between environmental exposures and ADHD risk but have little familiarity with the disorder’s diagnosis and prevalence, the functional domains that are impaired, or the underlying changes in brain structure and function. A second goal is to summarize behavioral deficits that are hallmarks of ADHD in order to facilitate comparisons with behavioral deficits associated with widely dispersed environmental chemicals—specifically lead and polychlorinated biphenyls (PCBs), which are reviewed in the companion paper by Eubig et al. (2010). At present, there is compelling evidence suggesting that several key brain functions are implicated in ADHD—attention, executive functions, processing of temporal information, and responses to reinforcement (Nigg and Nikolas 2008)—all of which are critical for modulating behavior (Barkley 1997; Nigg and Casey 2005). We review several meta-analyses published since 2004 that compare the performance of children and adolescents diagnosed with ADHD against non-ADHD controls on neuropsychological tasks measuring attention and executive functions. Additionally, we summarize the performance of ADHD children and adolescents on tests of temporal information processing and responses to reinforcement, which have not been evaluated in meta-analyses to date.

Meta-analyses were obtained through searches of PubMed (http://www.ncbi.nlm.nih.gov/pubmed/) using the terms “ADHD,” “meta-analysis,” “attention,” “executive,” and “neuropsychological functions,” among others. Meta-analytic studies were included if they originated in 2004 or later, included children or adolescents, and measured the effect size of the association between neuropsychological deficits and ADHD in terms of Cohen’s *d*, which is a metric that is discussed ahead. If no meta-analysis was available for a particular neuropsychological function, nonquantitative reviews were included.

## ADHD Prevalence and Diagnostic Criteria

ADHD is characterized by impulsivity and inattention, has an onset in early school age, and can persist into adulthood, although the prevalence lessens with age (Faraone et al. 2006). The pooled worldwide prevalence of ADHD in children and adolescents is 5.29%, with a range of about 5–10% when children are considered alone and about 2.5–4% when adolescents are considered by themselves (Polanczyk et al. 2007). Among adults, the pooled prevalence of ADHD is 2.5% (Simon et al. 2009). Estimates of rates for ADHD persistence into adulthood vary depending on the definition of ADHD persistence. When only those meeting the full criteria for ADHD are considered, persistence rates are lower, around 15% at 25 years of age, whereas when cases of ADHD in partial remission are considered, rates climb to around 65% at 25 years of age (Faraone et al. 2006). Using retrospective self-reports, Kessler et al. (2005) found that ADHD persisted into adulthood in about 36.3% of cases.

One of the challenges with ADHD is the great heterogeneity of symptoms among affected children (Nigg 2006b; Nigg and Nikolas 2008; Nigg et al. 2006). The most common clinical scale for diagnosing ADHD, the scale in the *Diagnostic and Statistical Manual of Mental Disorders*, 4th edition (*DSM*-*IV*), Text Revision (American Psychiatric Association 2000), consists of 18 behavioral items and distinguishes among three ADHD subtypes (see Appendix). A predominantly inattentive type (ADHD-PI) is diagnosed when at least six items are selected from the inattentive-disorganized dimension; a predominantly hyperactive–impulsive type (ADHD-PH) is diagnosed when at least six items are selected from the hyperactive–impulsive dimension; and a combined type (ADHD-C) is diagnosed when at least six items are selected from each of the two dimensions. Behavioral symptoms listed in the scale are selected only if they occur often, have persisted for the preceding 6 months, and are maladaptive and incongruent with the individual’s developmental level. Additionally, an ADHD diagnosis is given only if at least some of the behavioral symptoms were present before 7 years of age, happen in more than one setting, cause clear and significant impairment in social, school, or work functioning, and do not happen in the course of another mental disorder.

Children with ADHD-C make up most clinical referrals, which could explain why some authors have noted that most research has focused on this ADHD subtype (Nigg 2006a; Nigg and Nikolas 2008). ADHD-PI tends to be more prevalent in girls (Nigg and Nikolas 2008), whereas ADHD-C is most frequently diagnosed in boys. Like many other childhood-onset behavioral disorders, ADHD is diagnosed more frequently in boys than in girls (Pastor and Reuben 2002; Polanczyk et al. 2007).

ADHD often co-occurs with one or more other *DSM*-*IV* disorders. Young (2008) estimates that up to two-thirds of ADHD children have one or more coexisting disorders. The most common disorders co-occurring with ADHD-C in boys in the large, multisite study of ADHD, the Multimodal Treatment Study of Children with ADHD (MTA) (National Institute of Mental Health 1999), were oppositional defiant disorder (> 32%), anxiety (> 22%), and conduct disorder (> 7%). According to Young (2008), anxiety disorders seem to be even more common in girls (~ 33%) than in boys, when ADHD children 6–17 years of age are considered. Depression and bipolar disorders are also common comorbidities among adolescents with ADHD, as are substance use disorders (Spencer 2006; Spencer et al. 2007; Young 2008). Other comorbidities that are less common among ADHD adolescents are eating disorders, sleep disorders, learning disabilities, and certain medical conditions such as tic disorders, epilepsy, and celiac disease (Young 2008). Comorbidity is another challenging factor in interpreting ADHD data and evaluating theoretical claims about underlying mechanisms.

## Neuropsychological Functions Affected in ADHD

### Attention

Attention is a multidimensional construct (Stefanatos and Baron 2007) that can be broadly defined as the facilitated processing of one piece of information over others (Nigg and Nikolas 2008). Attention consists of several interrelated processes including alertness and vigilance (Oken et al. 2006; Posner 1995). In psychology and cognitive neuroscience the term “alertness” is described as the ability to obtain an alert state by focusing rapidly on new or unexpected information or stimuli (Nigg and Nikolas 2008). Similarly, vigilance or sustained attention is described as the ability to maintain attention on a task for a period of time once the alert state is entered (Oken et al. 2006).

Research indicates that children with ADHD have problems with alertness as well as with vigilance. These two attentional functions can both be assessed with continuous performance tasks (CPTs), which measure the ability to respond to a rare target (e.g., the letter “X” when it is preceded by the letter “A” but not by other letters) over an extended period of time (usually ≥ 15 min).

Table 1 lists the two attention functions that are impaired in ADHD individuals, the neuropsychological tasks used to assess the functions, the behavioral findings obtained with ADHD individuals, and meta-analyses that estimated the strength of association between deficits in these functions and ADHD based on Cohen’s *d*, which is defined as the difference in means divided by the pooled standard deviation across study populations. Cohen’s *d* is a standardized measure often used to compare the effects of variables measured on different scales and to estimate effect size across different studies. Cohen (1988) categorizes effect sizes around 0.2 as small, around 0.5 as moderate, and around 0.8 as large. Meta-analyses that focused only on ADHD adults, were published before 2004, or did not measure effect sizes in terms of Cohen’s *d* are not included.

#### Alertness

Alertness can be measured by the subject’s reaction time or how quickly the individual responds to the target stimuli (Posner 1995). Based on a meta-analysis of 13 studies of CPT performance in individuals diagnosed with ADHD, Frazier et al. (2004) reported that those with an ADHD diagnosis were slower than non-ADHD controls in responding to the target, with a small to moderate pooled effect size across studies (*d* = 0.39) (Table 1). Slower reaction times in ADHD children are not constrained to CPT tasks. For example, a recent study (Albrecht et al. 2008) compared boys diagnosed with ADHD with their unaffected siblings and with non-ADHD controls using a computerized reaction time task in which the stimuli were either congruent or incongruent with a previous target stimulus. ADHD boys were slower in their correct responses on both congruent and incongruent trials than were the non-ADHD controls. Interestingly, the unaffected siblings of the ADHD boys were midway between the two other groups; they did not differ significantly from either their ADHD siblings or the controls.

#### Vigilance

Vigilance is commonly assessed by errors of omission (misses) on CPTs. Two meta-analyses, one of 30 and the other of 33 studies that were published in 2004 or later, found that on CPTs, ADHD children made more errors of omission than non-ADHD controls did (Frazier et al. 2004; Willcutt et al. 2005). Both meta-analyses reported moderate effect sizes. The two meta-analyses did not employ completely unique data sets. Unfortunately, not enough information was available in Frazier et al. (2004) to ascertain the degree of overlap.

### Executive functions

Executive function refers to a set of abilities including working memory, response inhibition, and error correction that are involved in goal-directed problem solving (Marcovitch and Zelazo 2009). Executive function allows an individual to plan a series of steps necessary to achieve a desired goal, keep these steps in mind while acting on the goal, monitor progress through these steps, and have the cognitive flexibility to adjust or change the steps if progress is not being made toward the original goal. Table 2 lists the executive functions that have been identified as impaired in a number of meta-analytic studies of ADHD children and adolescents published since 2004 (Alderson et al. 2007; Frazier et al. 2004; Homack and Riccio 2004; Lansbergen et al. 2007; Lijffijt et al. 2005; Martinussen et al. 2005; Romine et al. 2004; van Mourik et al. 2005; Walshaw et al. 2010; Willcutt et al. 2005). Table 2 includes the neuropsychological tasks commonly used to assess these functions, the behavioral findings obtained with ADHD individuals, and the strength of the association, with the resulting effect sizes expressed as Cohen’s *d*. The inclusion criteria for Table 2 are similar to those for Table 1: The table lists only meta-analyses published since 2004 that reported effect sizes as Cohen’s *d* and that analyzed studies whose samples included children. As in Table 1, there is overlap in the studies included in the various meta-analyses in Table 2.

#### Working memory

Working memory is the ability to hold something in mind momentarily while doing something else or while using the information to perform an action (Baddeley 1986). Research indicates that there are separate neural circuits for working memory processes that involve verbal information (verbal working memory) versus spatial information (spatial working memory) (Baddeley 1996). Myriad neuropsychological tasks index verbal and spatial working memory function. Since 2004, three meta-analyses (Martinussen et al. 2005; Walshaw et al. 2010; Willcutt et al. 2005) evaluated studies on working memory in ADHD children and adolescents. These studies found moderate effect sizes ranging from 0.55 to 0.63 for impairments in ADHD children and adolescents compared with non-ADHD controls on seven different verbal working memory tasks: Digits Backward, Sentence Span, Color/Digit Span, Children’s Memory Scale Numbers Backward, Counting Span, Paced Auditory Serial Addition Task, and Self-Ordered Pointing Task (SOPT)-Objects. Table 2 gives short descriptions of each of these tasks. Larger effect sizes ranging from 0.63 to 1.04 were observed for impairments in children and adolescents diagnosed with ADHD compared with non-ADHD controls in five spatial working memory tasks: spatial span, a spatial working memory task from the Cambridge Neuropsychological Test Automated Battery (CANTAB); Finger Windows Test Backward; SOPT-Abstract; and the Spatial Span Backward task from the Wechsler Adult Intelligence Scale (WAIS). Table 2 also provides brief descriptions of these spatial working memory tasks.

#### Response inhibition

Response inhibition refers to the ability to inhibit or interrupt a response during dynamic moment-to-moment behavior (Nigg and Nikolas 2008). Key paradigms that tap this ability and have shown significant deficits in ADHD children are the go/no-go task, the stopping or stop signal time (SST) task (Aron and Poldrack 2005; Winstanley et al. 2006), the fixed interval schedule of reinforcement (Sagvolden et al. 1998), and CPTs. Only meta-analyses of studies of response inhibition in SST and CPT tasks met the criteria for inclusion in Table 2, so this discussion focuses on these two response inhibition measures.

As Huizenga et al. (2009) describe, in the SST task subjects are typically required to make rapid choice responses to “go” signals (e.g., press a button with the right hand if they see an X and a button with the left hand if they see an O). At random and occasional time intervals, a stop signal (e.g., the letter A or a tone) is presented shortly after the go signal, instructing the subject to inhibit the already initiated response activated by the go signal. As listed in Table 2, since 2004, five meta-analyses (Alderson et al. 2007; Frazier et al. 2004; Lijffijt et al. 2005; Walshaw et al. 2010; Willcutt et al. 2005) estimated Cohen’s *d* effect size for SST studies that included or were limited to children. The analyses indicated that, compared with non-ADHD individuals, those diagnosed with ADHD were consistently slower in stopping an ongoing response, suggesting difficulty in response inhibition. Effect sizes for stop signal reaction times in ADHD samples were in the moderate range (*d* = 0.54–0.63).

Commission errors (or false alarms) in CPTs are also often used as a marker of response inhibition deficits in ADHD children. Since 2004, three meta-analyses (Frazier et al. 2004; Walshaw et al. 2010; Willcutt et al. 2005) have examined the strength of the association between CPT commission errors and ADHD diagnosis in studies that included children and teens and calculated Cohen’s *d* effect sizes (Table 2). As in the SST analyses, the results for CPT commission errors were in the moderate range (*d* = 0.51–0.56).

#### Cognitive flexibility

The ability to switch attention from one aspect of an object to another, or to adapt and shift one’s response based on situational demands, such as changes in the rules, schedule, or type of reinforcement in a task, is defined as cognitive flexibility or set shifting (Monsell 2003; Stemme et al. 2007). Tests used to assess cognitive flexibility in children include the Wisconsin Card Sorting Test (WCST), the Stroop Color-Word test (Stroop task), and the Trail Making Test Part B (Trails-B).

On the WCST, subjects are asked to sort into two different piles a series of cards with figures that can differ in color, shape, and/or number. Each time a card is sorted, the subject receives feedback as to whether the choice was correct or incorrect, and based on this feedback the subject must infer the correct category (color, shape, or number) for sorting (Romine et al. 2004). After the subject correctly sorts the cards in a series of consecutive trials, the sorting category is changed and the subject must learn the new sorting category by trial and error. An indicator of impairments in cognitive flexibility is the tendency to make perseverative errors or persist in sorting the cards by the previously correct category, even after being told the sorting strategy is incorrect. Four recent meta-analyses (Frazier et al. 2004; Romine et al. 2004; Walshaw et al. 2010; Willcutt et al. 2005) computed small (0.35) to medium (0.52) effect sizes for the differences in mean perseverative errors between ADHD individuals and non-ADHD controls on the WCST (Table 2). ADHD individuals made more perseverative errors on the WCST than did non-ADHD controls, suggesting that ADHD is associated with impaired cognitive flexibility.

In the Stroop task, problems in cognitive flexibility are measured by the degree of difficulty subjects have in naming the color of the ink used to print color words when the two are mismatched (e.g., when the word “green” is printed in blue ink). Interference scores quantify subjects’ difficulty in the task, with higher scores indicating greater difficulty. Effect sizes for Stroop interference scores reported in five recent meta-analyses (Frazier et al. 2004; Homack and Riccio 2004; Lansbergen et al. 2007; van Mourik et al. 2005; Walshaw et al. 2010) vary widely from small (0.35) to large (1.11), making it hard to characterize the findings (Table 2). This inconsistency may be at least partially due to variation in the method used to calculate the interference score across studies. [For a description of different ways of deriving interference scores, see Homack and Riccio (2004).]

Another widely used tool for assessing cognitive flexibility is Trails-B, in which subjects are presented with numbers and letters inside circles that are randomly arranged on a sheet of paper. Subjects are asked to connect in ascending order the numbers and letters while alternating between them (e.g., 1–A–2–B–3–C–4); they are asked to do this as quickly as possible (Lezak et al. 2004). Time to complete the task is measured, with longer response times indicative of difficulties in cognitive flexibility. Two meta-analyses (Frazier et al. 2004; Willcutt et al. 2005) have reported medium effect sizes (*d* = 0.55 and 0.59 respectively, as shown in Table 2) as evidence of reduced cognitive flexibility in ADHD versus control children based on Trails-B scores.

#### Planning

Some researchers have found that deficits in planning and strategy development discriminate well between children with ADHD and those without (Papadopoulos et al. 2005). ADHD children have been found to perform poorly in four tasks that are commonly used to assess planning ability: Tower of Hanoi (TOH) task and its variant the Tower of London (TOL) task, Porteus Maze, and Rey-Osterrieth Complex Figure Task (ROCF).

Tower tasks such as TOH and TOL are a popular neuropsychological measure of planning (Riccio et al. 2004). The many variations of this task basically involve moving stacked beads or disks of different sizes to new positions that match the model provided. This must be accomplished in a minimum number of moves and while following rules for moving the objects (e.g., only one disk can be moved at a time, no disk can be placed on top of a smaller disk) (Papadopoulos et al. 2005; Riccio et al. 2004). It is assumed that subjects will generate a more efficient solution if they plan a series of moves before actually beginning to move the beads or disks (Riccio et al. 2004).

In the Porteus Maze task, subjects are presented with mazes of increasing difficulty. They must find a solution (i.e., the way out) while following a number of rules (e.g., no entering a dead end, no backtracking) (Levin et al. 2001). Planning the movement through the maze increases the subjects’ ability to adhere to the rules. In the ROCF, individuals are asked to copy and later recall a complex figure composed of 64 segments. In both stages the examiner can rate the accuracy of the different lines as well as the level of organization when clustering lines during the copying and recall phases (Sami et al. 2003). Higher levels of organization are indicative of better strategic planning. Three recent meta-analyses (Frazier et al. 2004; Walshaw et al. 2010; Willcutt et al. 2005) indicate effect sizes in the low to medium range (*d* = 0.24–0.69) (Table 2) for the differences between ADHD individuals and non-ADHD controls in these four planning tasks.

### Summary of meta-analytic studies

In summary, meta-analyses indicate that performance is impaired in ADHD individuals on a large number of attention and executive function tasks. Within the attention and executive function domains, larger deficits are found on tasks measuring vigilance, working memory (especially spatial working memory), and response inhibition abilities, whereas smaller but significant deficits are also seen on tasks measuring alertness, cognitive flexibility, and planning abilities. There is overlap in the studies included in some of the meta-analyses discussed herein. Thus, the individual analyses cannot be taken as totally independent indicators of the effect. Also, deficits on any single test of attention or executive function are not sufficient for a diagnosis of ADHD (e.g., Homack and Riccio 2004) or for differentiating ADHD from other mental or learning disorders (e.g., Walshaw et al. 2010). This should not be surprising given the great heterogeneity of symptoms across affected individuals. Finally, meta-analyses to date have lacked in-depth analyses of the associations between patterns of behavioral deficits on the various neuropsychological tasks and the three different ADHD diagnoses (ADHD-C, ADHD-PI, and ADHD-PH), primarily because most ADHD studies, especially older studies, have not evaluated ADHD subtypes.

### Temporal information processing and responses to reinforcement

Two other types of deficits related to the processing of temporal information and to responses to the reinforcing properties of rewards have been reported in ADHD children but have not been subjected to meta-analysis. These deficits could contribute to the difficulties ADHD children have in executive function tasks. Recent studies have focused increasingly on temporal information processing, which is believed to be key to the control and modulation of behavior (Barkley 1997; Nigg and Casey 2005). Toplak et al. (2006) reviewed 38 studies that measured temporal information processing in ADHD children. Most of these studies used tasks in which the child was asked to indicate the end of a specific time interval, either by holding down a response key for the specified interval or by responding verbally to indicate the end of the interval. There were no external cues by which the child could estimate the interval. Most studies found poor time estimation in children with ADHD, especially when longer time intervals were employed.

In terms of responses to reinforcement, Luman et al. (2005) reviewed 22 studies comparing the responses of children with and without an ADHD diagnosis to reinforcement contingencies in a variety of tasks. The authors concluded that ADHD is associated with increased weighting of near-term over long-term (but larger) rewards, positive response to high-intensity reinforcement, and a lack of a physiological response, such as heart rate acceleration, to potential rewards. The pattern of results in these studies suggests that ADHD children have difficulty reasoning about rewards and, as a result, do not respond appropriately to reinforcements. Although abnormalities in responses to reinforcement have been studied in the context of motivation, they could be related to impairments in executive functioning, especially in the case of difficulties in weighing near-term versus long-term rewards.

## Neural Imaging Studies of ADHD Patients

**Figure 1:**
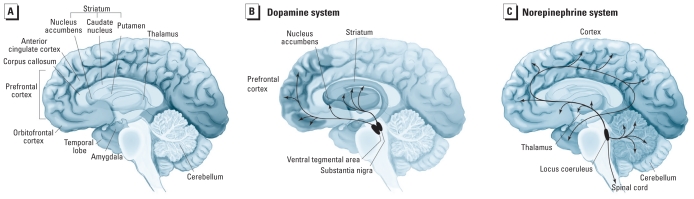
Neuroanatomical structures and dopaminergic and noradrenergic neuronal projections that have roles in ADHD. The illustrations are of the medial surface of a hemisected human brain. (*A*) Reductions in prefrontal cortical, caudate nucleus, corpus callosum, and cerebellar volumes are seen in ADHD. Altered functioning of the anterior cingulate and orbitofrontal cortices, the amygdala, and the nucleus accumbens has also been demonstrated in ADHD. The striatum includes the caudate nucleus, the putamen, and the nucleus accumbens. (*B*) Dopaminergic neurons that are important in ADHD arise in the ventral tegmental area of the midbrain and project to the frontal cortical and limbic structures, where they serve to modulate neurochemical signaling. Other dopaminergic neurons arise from the substantia nigra and project to the striatum, where they participate in controlling voluntary movement. (*C*) Noradrenergic neurons arise from the locus coeruleus and project to numerous structures including the prefrontal cortex, the limbic system, the thalamus, and the cerebellum. Adapted from Bear et al. (2001).

The heterogeneity in symptoms and functional deficits observed in ADHD is paralleled by heterogeneity in the results of brain imaging studies. Although many individuals with ADHD do not have abnormal structural magnetic resonance imaging (MRI) results, when the results are considered across individuals in an ADHD sample, a pattern of structural changes becomes evident (Nigg and Nikolas 2008). Overall, there is a reduction of up to 5% in brain volume, with greater reductions in the prefrontal cortex, caudate nucleus, cerebellum, and corpus callosum (Nigg and Nikolas 2008; Valera et al. 2007) (Figure 1A). Smaller brain volume tends to be associated with a greater severity of ADHD symptoms (Krain and Castellanos 2006).

There is strong evidence for altered corticostriatal circuitry in ADHD. This circuit includes the dorsolateral prefrontal and dorsoanterior cingulate cortices, the dorsal striatum (especially the caudate nucleus), and the thalamus, which links to the cerebellum (Sonuga-Barke 2005; Vaidya and Stollstorff 2008). The dorsolateral prefrontal cortex has roles in planning and organizing behavior, working memory, and response inhibition (Nigg and Nikolas 2008). The anterior cingulate cortex has roles in cognition and motor control and is specifically involved in processes underlying the arousal/drive state of the organism (Makris et al. 2009). The dorsal striatum plays an important modulatory role in controlling responses (Nigg and Nikolas 2008), whereas the cerebellum is important for coordinating motor activities as well as timing and shifting attention (Krain and Castellanos 2006).

Bilateral prefrontal cortices, the right caudate, and regions of the cerebellum were all found to be reduced in size in a meta-analysis of structural MRI findings (Valera et al. 2007), whereas the left dorsolateral prefrontal and anterior cingulate cortices, right caudate, and right thalamus were shown to be hypoactive in a meta-analysis of functional MRI data from ADHD individuals performing tests of executive functioning (Dickstein et al. 2006).

A limited number of functional MRI studies suggest alterations in functional connections between components of the corticolimbic circuit (Vaidya and Stollstorff 2008). This circuit includes the orbitofrontal and anterior cingulate cortices, the ventral striatum (especially the nucleus accumbens), the thalamus, and regions of the amygdala (Sonuga-Barke 2005; Vaidya and Stollstorff 2008) (Figure 1). The orbitofrontal cortex integrates sensory and affective information as part of reward processing, whereas the ventral striatum has roles in reward-related emotion and motivation (Fareri et al. 2008).

## Neurochemistry of ADHD

Converging lines of evidence argue that dysfunctional catecholaminergic signaling underlies the cognitive alterations seen with ADHD (Vaidya and Stollstorff 2008). The prefrontal cortex receives both dopaminergic and noradrenergic innervation, whereas the striatum has generous dopaminergic innervation but sparse noradrenergic innervation (Figure 1B,C). In these regions, both of which are implicated in ADHD, catecholaminergic systems modulate glutaminergic and GABAergic (γ-aminobutyric acid) neurotransmitter release (Brennan and Arnsten 2008). Catecholaminergic transporters, including both dopamine and norepinephrine transporters, exert an important influence on dopamine neurotransmission in the prefrontal cortex and striatum.

Although the exact nature of the neurochemical deficits underlying ADHD is still unknown, there is evidence that hypoactivity of frontostriatal dopamine circuits (reviewed by Swanson et al. 2007) and abnormal noradrenergic signaling (Brennan and Arnsten 2008) play a role. Imaging studies have identified apparent increases of dopamine transporter and dopamine D2 receptor numbers in ADHD patients (Nikolaus et al. 2007), although a recent study in medication-naive ADHD adults found decreases in dopamine transporter and dopamine D2/3 receptors (Volkow et al. 2009). Finally, the improvements in symptoms seen with medications that target catecholaminergic systems indirectly suggest dysfunctional dopaminergic signaling in ADHD. Effective pharmacotherapies for ADHD include stimulant medications, such as methylphenidate and amphetamine, which increase synaptic dopamine release (Madras et al. 2005; Pliszka 2005). Other beneficial medications include the norepinephrine transporter inhibitor atomoxetine, which inhibits the reuptake of dopamine in the prefrontal cortex, and the α2A agonist guanfacine, which increases delay-related firing in the prefrontal cortex (Brennan and Arnsten 2008; Madras et al. 2005; Pliszka 2005).

ADHD cannot be explained by simple deficiencies or excesses of synaptic catecholamines (Pliszka 2005). Alterations in the interactions between neurotransmitter systems are likely to better explain ADHD. Also, the relative levels of monoamines (including serotonin) may be more important than absolute levels (Winstanley et al. 2006). However, these ambiguities should not distract from the large body of evidence that implicates alterations in dopaminergic and noradrenergic signaling as important underlying factors in the pathogenesis of ADHD.

## Genetics of ADHD

ADHD is a highly heritable disorder based on findings from family, twin, and adoption studies. The risk of ADHD in parents and siblings of children with ADHD is increased two to eight times (Franke et al. 2009), with heritability estimated at 76% based on pooled data from twin studies (Franke et al. 2009; Smith et al. 2009). Hence, much effort has focused on genetic studies of ADHD.

Candidate gene studies focus on specific genes identified *a priori* as important in neurotransmitter pathways relevant to ADHD (Brookes et al. 2006; Nigg and Nikolas 2008). Polymorphisms in the dopamine transporter gene (*DAT1*, *SLC6A3*) and the dopamine 4 (D4) receptor gene (*DRD4*) have been most often associated with ADHD; other candidate genes with significant associations in meta-analyses include the dopamine D5 receptor (*DRD5*), serotonin transporter (*5HTT*, *SLC6A4*), serotonin receptor 1B (*5HT1B*, *HTR1B*), and synaptosomal-associated protein 25 (*SNAP25*) (Gizer et al. 2009; Smith et al. 2009). Polymorphisms in the norepinephrine transporter gene (*NET1*, *SLC6A2*) also have been associated with ADHD (e.g., Brookes et al. 2006; Kim et al. 2008), although meta-analytic findings have not been strong for *NET1*. Overall, the associations from candidate gene studies have been very modest, with no gene accounting for > 3–4% of the total variance in ADHD phenotype (Smith et al. 2009).

Genomewide linkage scans, which are family based, and genomewide association studies (GWAS), which are population based, differ from candidate gene studies in that the entire genome is analyzed without *a priori* hypotheses (Franke et al. 2009). These approaches can suggest novel genes that may be involved in the pathogenesis of ADHD. Although genomewide linkage scans have identified chromosome regions that might contain genes associated with ADHD (reviewed by Smith et al. 2009), the findings have not replicated well across studies (Zhou et al. 2008). This may be attributable partly to the fact that linkage studies are best able to identify polymorphisms that account for ≥ 10% of the phenotypic variance of a disorder (Franke et al. 2009). The absence of significant findings from genomewide linkage studies suggests that the effects of DNA risk variants are individually very small despite the high heritability of ADHD (Faraone et al. 2008). In line with this, a recent meta-analysis of seven ADHD genomewide linkage studies identified a significant signal on chromosome 16, whereas none of the individual studies was able to detect a signal at that location (Zhou et al. 2008), suggesting that combining individual studies to increase power may be a valuable approach.

GWAS is a more powerful, unbiased method used to search for risk genes of smaller effect (Psychiatric GWAS Consortium Coordinating Committee et al. 2009). So far, GWAS of ADHD has produced a limited number of significant findings and little overlap between studies (Banaschewski et al. 2010; Franke et al. 2009). However, genes related to cell–cell communication and adhesion, neuronal migration, and potassium-related signaling are commonly found in the top ADHD GWAS rankings, suggesting candidate genes for further study (Banaschewski et al. 2010; Franke et al. 2009). Much remains to be understood about the genetic causes of ADHD. However, GWAS with greater sample size is under way, which, when combined with meta-analytical approaches, holds much promise for further elucidating the genetics of ADHD.

## Conclusion

ADHD is a complex disorder with great heterogeneity in the behavioral symptoms presented and brain functions and structures affected. It is clear, however, that several aspects of attention and executive function—particularly vigilance, working memory, and response inhibition—are compromised in ADHD children. Deficits in the processing of temporal information and the processing of rewards are also associated with ADHD and could be either related to or exacerbated by the deficits in executive function.

Although much research has been done on the neuropsychological, neuroanatomical, neurochemical, and genetic bases of ADHD, we are still far from fully understanding its etiology. Given the inability to explain ADHD on a solely genetic basis, interest in the contribution of environmental factors—including exposure to chemical contaminants—has intensified. To date, most of what has been written on this topic focuses on just two contaminants, lead and PCBs, although potential contributions of other chemicals are beginning to be explored. In our companion review (Eubig et al. 2010), we discuss evidence for effects of lead and PCBs on the components of attention and executive function that are impaired in ADHD children. It is our hope that by highlighting the parallels between the neurobehavioral effects of these contaminants and the deficits observed in ADHD children, we will motivate further research on the contribution of environmental chemical exposures to ADHD.

## Figures and Tables

**Table 1:** Attention functions impaired in ADHD: meta-analyses of studies comparing ADHD and control.

Attention function	Task name and description	Behavioral finding^a^	No. of studies in meta-analysis (*k*)	ADHD subjects summed across studies (*n*)	Age range^b^	Effect size (Cohen’s *d*)	Reference
Alertness	CPT: Latency to respond to target sequence is the hit RT; its SE indicates the consistency in focusing attention	↑ SE hit RT	13	NA	Children–adult	0.39	Frazier et al. 2004
Vigilance	CPT: Respond rapidly to target sequence; failure counts as omission error	↑ omission errors	33 30	NA 1,366	Children–adult Children–teens	0.66 0.64	Frazier et al. 2004 Willcutt et al. 2005

Abbreviations: CPT, continuous performance task; NA, not available; RT, reaction time; SE, standard error.

a ↑ indicates significant increase associated with ADHD.

b Age range is for all studies examined in the referenced article; an age breakdown was not given for the individual neuropsychological tasks included in the meta-analyses.

**Table 2:** Executive functions impaired in ADHD: meta-analyses of studies comparing ADHD and control.

Executive function/task name and description	Behavioral finding^a^	No. of studies in meta-analysis (*k*)	ADHD subjects summed across studies (*n*)	Age range	Effect size (Cohen’s *d*)	Reference
Verbal working memory						

DB: repeat series of numbers in reverse order of presentation	↓ digits recalled	7	548	Children–teens	0.63	Walshaw et al. 2010

SeS: generate final word missing in sentences, then recall all words generated	↓ words recalled	11	718	Children–teens^b^	0.55	Willcutt et al. 2005 (DB and SeS were analyzed together)

Color/Digit Span: recall items in order of presentation	↓ items recalled					See Martinussen et al. 2005

CMS Numbers-B: recall number sequence in reverse order	↓ items recalled					See Martinussen et al. 2005

Counting Span: count groups of shapes and remember count totals	↓ items recalled					See Martinussen et al. 2005

PASAT: add single digit numbers presented at varying speeds	↓ correct additions					See Martinussen et al. 2005

SOPT-Objects: select different familiar items across sets of items in different arrangements	↑ repeated selections	13	475	4–18 years	0.56	Martinussen et al. 2005 (all VWM tasks listed were analyzed together)

Spatial working memory						

SpS: mentally rearrange spatial configuration of blocks and produce a response	↓ blocks correct	3	61	Children–teens^b^	0.94	Walshaw et al. 2010

CANTAB SWM: remember where previously searched tokens were found to avoid revisiting these places (between-search error)	↑ between-search errors	7	292	Children–teens	0.77	Walshaw et al. 2010

SOPT-Abstract: same as SOPT-Objects, except items are abstract shapes	↑ repeated selections	8	342	Children–teens^b^	0.63	Willcutt et al. 2005 (SOPT-Abstract and CANTAB SWM analyzed together)

FWT-B: reproduce in reverse sequence of locations presented	↓ locations recalled					See Martinussen et al. 2005

WAIS SpS-B: reproduce in reverse sequence of blocks tapped by examiner	↓ locations recalled	8	161	Children–teens^b^	1.06	Martinussen et al. 2005 (CANTAB SWM, SOPT-Abstract, FWT-B, and WAIS SpS-B

Response inhibition						

SST: inhibit ongoing response	↑ RT when tone is heard	13	NA	Children–adult^b^	0.54	Frazier et al. 2004
		27	1,104	Children–teens^b^	0.61	Willcutt et al. 2005
		17	1,195	6–13 years	0.58	Lijffijt et al. 2005
		22	726	6–12 years	0.63	Alderson et al. 2007
		25	1,054	Children–teens	0.63	Walshaw et al. 2010

CPT: inhibit response to nontarget sequence, failure counts as commission error	↑ commission errors	40	NA	Children–adult^b^	0.55	Frazier et al. 2004
		28	1,390	Children–teens^b^	0.51	Willcutt et al. 2005
		23	994	Children–teens	0.56	Walshaw et al. 2010

Cognitive flexibility						

WCST: sort picture/symbol cards according to shifting rules	↑ perseverative errors	25	NA	Children–adult^b^	0.35	Frazier et al. 2004
		21	NA	Children	0.52	Romine et al. 2004
		24	1,259	Children–teens^b^	0.46	Willcutt et al. 2005
		18	1,064	Children–teens	0.36	Walshaw et al. 2010

Stroop: name ink colors used to print color words; ink and color words are mismatched	↑ interference score	20	NA	Children–adult^b^	0.56	Frazier et al. 2004
		13	407	6–13 years	0.58	Homack and Riccio 2004
		17	1,395	6–27 years	0.35	van Mourik et al. 2005
		15	817	Children–teens	0.35	Walshaw et al. 2010
		7	148	7–47 years	1.11	Lansbergen et al. 2007 (only time-per-item studies that do not use Golden’s method)

Trails-B: connect letters and numbers in ascending order while alternating between them	↑ response time	14	NA	Children–adult^b^	0.59	Frazier et al. 2004
		14	609	Children–teens^b^	0.55	Willcutt et al. 2005

Planning						

TOL/TOH: move stacked objects to new position while following rules on how to move them	↓ score	6	186	Children–teens^b^	0.69 (TOH)	Willcutt et al. 2005
		6	383	Children–teens^b^	0.51 (TOL)	Willcutt et al. 2005
		7	373	Children–teens	0.38 (TOL)	Walshaw et al. 2010

PM: exit maze w/ no backtracking	↓ score	5	324	Children–teens^b^	0.58	Willcutt et al. 2005

ROCF: copy an abstract figure score	↓ organization score	6	NA	Children–adult^b^	0.24	Frazier et al. 2004
		9	587	Children–teens	0.43	Willcutt et al. 2005

Abbreviations: CANTAB, Cambridge Neuropsychological Test Automated Battery; CMS, Children’s Memory Scale; CMS Numbers-B, Children’s Memory Scale Numbers Backward; DB, Digits Backward; FWT-B, Finger Windows Test Backward; NA, not available; PASAT, Paced Auditory Serial Addition Task; PM, Porteus Maze; ROCF, Rey-Osterrieth Complex Figure Task; RT, reaction time; SeS, sentence span; SOPT, Self-Ordered Pointing Task; SpS, spatial span; SpS-B, Spatial Span Backward; SST, stop signal time; Stroop, Stroop Color-Word test; SWM, spatial working memory; Trails-B, Trail Making Test Part B; TOH, Tower of Hanoi; TOL, Tower of London; VWM, verbal working memory; WAIS, Wechsler Adult Intelligence Scale; WCST, Wisconsin Card Sorting Test.

a ↑ indicates significant increase associated with ADHD; ↓ indicates significant decrease.

b Age range is for all studies examined in the referenced article; an age breakdown was not given for the individual neuropsychological tasks included in the meta-analyses.

## Appendix I Diagnostic Criteria for ADHD^a^

- At least six behavioral symptoms from list A or list B occur often, have persisted for the preceding 6 months, and are maladaptive and inappropriate given the individual’s developmental level.
  - Inattentive–Disorganized Dimension:
    1. Fails to give close attention to details or makes careless mistakes in schoolwork, work, or other activities
    2. Has difficulty sustaining attention in tasks or play activities
    3. Does not seem to listen when directly spoken to
    4. Fails to follow through on instructions and fails to finish schoolwork, chores, or work duties
    5. Has difficulty organizing tasks and activities
    6. Avoids, dislikes, or is reluctant about engaging in tasks that require sustained mental effort
    7. Loses things necessary for tasks or activities (e.g., toys, school assignments, or tools)
    8. Gets easily distracted by extraneous stimuli
    9. Is forgetful in daily activities
  - Hyperactivity–Impulsivity Dimension:
    1. Fidgets with hands or feet or squirms in seat
    2. Leaves seat in classroom or in other situations in which remaining seated is expected
    3. Runs about or climbs excessively in situations in which it is inappropriate (in adolescents or adults, may be limited to subjective feelings of restlessness)
    4. Has difficulty playing or engaging in leisure activities quietly
    5. Is “on the go” or acts as if “driven by a motor”
    6. Talks excessively
    7. Blurts out answers before questions have been completed
    8. Has difficulty awaiting turn
    9. Interrupts or intrudes on others (e.g., butts into conversations or games)
- Some symptoms that cause impairment were present before 7 years of age.
- Some impairment from the symptoms is present in two or more settings.
- There is clear evidence of significant impairment in social, school, or work functioning.
- Symptoms do not happen only during the course of a pervasive developmental disorder, schizophrenia, or other psychotic disorder, and they are not better accounted for by another mental disorder (e.g., mood, anxiety, dissociative, or personality disorder).

Based on criteria I–V, three types of ADHD are identified:

1. Predominantly inattentive (ADHD-PI): if at least six symptoms from list A but not B are present
2. Predominantly hyperactive–impulsive (ADHD-PH): if at least six symptoms from list B but not A are present
3. Combined (ADHD-C): if at least six symptoms from each of the lists, A and B, are present
